# Co-Encapsulation of Mitoxantrone and β-Elemene in Solid Lipid Nanoparticles to Overcome Multidrug Resistance in Leukemia

**DOI:** 10.3390/pharmaceutics12020191

**Published:** 2020-02-23

**Authors:** Kambere Amerigos Daddy J.C., Minglei Chen, Faisal Raza, Yanyu Xiao, Zhigui Su, Qineng Ping

**Affiliations:** State Key Laboratory of Natural Medicines, Department of Pharmaceutics, School of Pharmacy, China Pharmaceutical University, Nanjing 210009, China

**Keywords:** multidrug resistance, mitoxantrone, β-elemene, solid lipid nanoparticles, leukemia, P-glycoprotein

## Abstract

Multidrug resistance (MDR) due to P-glycoprotein (P-gp) overexpression is a major obstacle to successful leukemia chemotherapy. The combination of anticancer chemotherapy with a chemosensitizer of P-gp inhibitor is promising to overcome MDR, generate synergistic effects, and maximize the treatment effect. Herein, we co-encapsulated a chemotherapeutic drug of mitoxantrone (MTO) and a P-gp inhibitor of β-elemene (βE) in solid lipid nanoparticles (MTO/βE-SLNs) for reversing MDR in leukemia. The MTO/βE-SLNs with about 120 nm particle size possessed good colloidal stability and sustained release behavior. For the cellular uptake study, doxorubicin (DOX) was used as a fluorescence probe to construct SLNs. The results revealed that MTO/βE-SLNs could be effectively internalized by both K562/DOX and K562 cells through the pathway of caveolate-mediated endocytosis. Under the optimized combination ratio of MTO and βE, the in vitro cytotoxicity study indicated that MTO/βE-SLNs showed a better antitumor efficacy in both K562/DOX and K562 cells than other MTO formulations. The enhanced cytotoxicity of MTO/βE-SLNs was due to the increased cellular uptake and blockage of intracellular ATP production and P-gp efflux by βE. More importantly, the in vivo studies revealed that MTO/βE-SLNs could significantly prolong the circulation time and increase plasma half-life of both MTO and βE, accumulate into tumor and exhibit a much higher anti-leukemia effect with MDR than other MTO formulations. These findings suggest MTO/βE-SLNs as a potential combined therapeutic strategy for overcoming MDR in leukemia.

## 1. Introduction

Multidrug resistance (MDR) accounts for most chemotherapy-related failure in a wide array of malignancies. Mitoxantrone dihydrochloride (MTO: 1,4-dihydroxy-5, 8-bis[[2-[(2-hydroxyethyl) amino] ethyl] amino] anthraquinone dihydrochloride) was developed in the 1980s as a doxorubicin analogue in a program to find drugs with improved antitumor activity and decreased cardiotoxicity compared with the anthracyclines. The molecular formula of MTO is C_22_H_30_Cl_2_N_4_O_6_ and its molecular weight is 517.4 g/mol. Structurally, MTO is symmetrical, containing a tricyclic planar chromophore substituted with two nitrogen-containing side chains (Figure S1A, Supplementary Materials) [1]. MTO, a cell cycle non-specific anticancer drug and P-glycoprotein (P-gp) substrate, is a synthetic anthracenedione that is applied for the treatment of acute leukemia, malignant lymphoma [2,3], breast cancer [4], and hepatocellular carcinoma [5]. The underlying anticancer effects of MTO are credited to its binding affinity to DNA and the resultant inhibition of DNA topoisomerase II [6]. Previous studies reported reduced levels of MTO in leukemia cells overexpressing P-gp as a major limitation to its anticancer effect [7,8,9]. As part of efforts at nibbling this problem in the bud, co-delivery of anticancer drugs with ATP-binding cassette (ABC) transport inhibitors seems a viable strategy. By so doing, delivery of these drugs to tumor sites is markedly enhanced due to the inhibition of drug efflux transporters [10,11]. The numerous side effects of known P-gp inhibitors like verapamil and cyclosporine emphasize the need for better alternatives. β-Elemene (βE: 1-methyl-1-vinyl-2,4-diisopropenyl-cyclohexane), is isolated from the Chinese medicinal ginger plant *Rhizoma zedoariae* [12]. The molecular formula of βE is C_15_H_24_ and its molecular weight is 204.34 g/mol. The chemical structure of βE is shown in Figure S1B (Supplementary Materials) [13]. βE is reported to possess broad antineoplastic activity with proven efficacy against drug-resistant tumors [14,15], and it is approved by China’s State Food and Drug Administration [16]. It can not only inhibit the growth of tumors, but also improve the immune function of the body [17]. It inhibits drug efflux via direct interaction with ABC transporter proteins, and downregulates P-gp expression and MDR1 gene products [18,19,20]. Therefore, βE functions as a chemosensitizer to enhance the cytotoxic effects of chemotherapeutic drugs against many MDR cells such as KB-C2 resistant cells, MCF-7/ADR, MCF-7/DOC, K562/ADR, and K562/DNR [20,21,22,23,24]. Several studies showed combination therapy as a viable approach to militate against MDR, as well as enhance efficacy with minimal side effects by selectively killing resistant cells while protecting normal cells [25,26,27]. Miaobo et al. reported JL-17, a substitute of triazol-*N*-ethyl-tetrahydroisoquinoline, which is more potent against ABCB1 than verapamil and significantly increases the anticancer drug accumulation in K562/A02 cells. Furthermore, it was reported that ethyl lucidenates-A reverses 7.59-fold resistance of K562/A02 cells to vincristine [28]. To the best of our knowledge, there are no reports of βE combined with MTO so far.

**Scheme 1:**
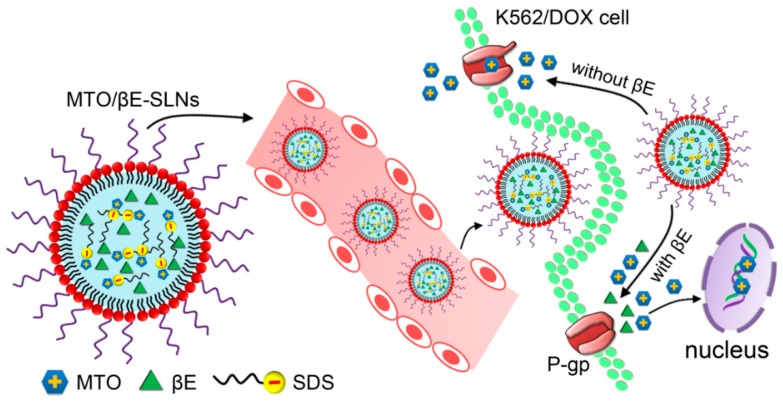
Schematic illustration of solid lipids nanoparticles for co-loading mitoxantrone (MTO) and β-elemene (βE) (MTO/βE-SLNs) to overcome multidrug resistance (MDR) in leukemia via P-glycoprotein (P-gp) inhibition. MTO/βE-SLNs are composed of an electrostatic complex of water-soluble cationic drug MTO and negative sodium dodecyl sulfate (SDS), βE, a solid lipid matrix, surfactants, and phospholipid.

In this study, MTO combined with βE was evaluated to reverse the MTO resistance in leukemia. Both MTO and βE were encapsulated into solid lipid nanoparticles (MTO/βE-SLNs) with the aim of enhancing solubility, extending circulation time, and improving desired effects, while decreasing the toxicity of MTO. After intravenous administration, MTO/βE-SLNs would recognize the leukemia cells during circulation time. After internalization into the leukemia cells, βE could function as a chemosensitizer for inhibiting P-gp, preventing the MTO efflux, and finally improving the anticancer effects (Scheme 1). To fulfill these goals, the synergistic anticancer effect of the combination of MTO and βE in different ratios (*w*/*w*) was evaluated. The key properties such as particle size, morphology, encapsulation efficiency (EE %), stability, and in vitro drug release of MTO/βE-SLNs were characterized. The cellular uptake and the MDR suppression efficiency of MTO/βE-SLNs in both leukemia cell line K562 and its multidrug resistant K562/DOX were investigated by using doxorubicin (DOX) as a fluorescence probe. In addition, drug efflux and intracellular adenosine triphosphate (ATP)-binding cassette production assays were determined to evaluate the reversal effect of MDR by MTO/βE-SLNs. Moreover, the in vivo effects and antitumor activity were investigated in K562/DOX xenografts tumor model mice.

## 2. Materials

### 2.1. Reagents

Soybean lecithin phospholipids (SPC, purity 90%) were purchased from Shanghai Taiwei Pharmaceutical (Shanghai, China). Poly(ethylene glycol)monostearate (S100) was obtained from Nanjing Well Pharmaceutical co., LTD. (Nanjing, China). Cholesterol (analytical grade) was provided by Biochemical Reagent Co., Ltd. (Shanghai, China). MTO (purity, 99.1%) was purchased from Sichuan Shenghe Pharmaceutical Co., Ltd. (Chengdu, Sichuan, China). βE (purity, 98.5%) was obtained from Shanghai Gaolang Chemical (Shanghai, China). DOX (purity, 98%), 3-(4,5-dimethylthiazol-2-yl)-2,5-diphenyltetrazoliumbromide (MTT), 1,1-dioctadecyl-3,3,3,3-tetramethylindotricarbocyanine iodide (DiR), dimethyl sulfoxide (DMSO), phosphate-buffered saline (PBS), and ATP assay kit were purchased from Nanjing KeyGen BioTECH (Nanjing, China). Trilaurin was obtained from Tokyo Chemical Industry (Tokyo, Japan). Kolliphor EL was provided by BASF Chemical Company (Ludwigshafen, Germany). Sodium dodecyl sulfate (SDS), chlorpromazine, nystatin, methyl beta cyclodextrin (M-β-CD), and amloride were purchased from Shanghai Aladdin Reagent (Shanghai, China). Methanol (HPLC grade), acetonitrile (HPLC grade), dichloromethane (analytical grade), phosphoric acid, ammonium acetate, Tween-80, and other reagents were obtained from Sinopharm Chemical Reagent Co., Ltd. (Shanghai, China).

### 2.2. Cell Cultures and Animals

Chronic myelogenous leukemia-derived K562 cells (sensitive) and K562/DOX cells (resistant) were purchased from Shanghai Gefan Biotechnology Co. Ltd. (Shanghai, China). K562 cells were cultured in Roswell Park Memorial Institute (RPMI)-1640 bicarbonate medium supplemented with 10% fetal bovine serum (FBS). Cells were incubated at 37 °C in a humidified atmosphere with 5% CO_2_. K562/DOX cells were grown in the same medium supplemented with 1 μg/mL DOX to maintain the resistant characteristics.

Sprague-Dawley rats (180–220 g) and BALB/c nude mice (18–20 g) were obtained from Shanghai Silaike Laboratory Co. Ltd. (Shanghai, China) and kept in the Animal Center of China Pharmaceutical University (Nanjing, China).They were housed in a room with controlled temperature and humidity and had free access to food and water. All animal experiments were performed in accordance with Provision and General Recommendation of Chinese Experimental Animals Administration Legislation and the Instructive Notions with Respect to Care for Laboratory Animals, and they were approved by the Animal Ethics Committee of China Pharmaceutical University and the Department of Science and Technology of Jiangsu Province (license number: SYXK(SU)2016-0011; 27 January 2016).

## 3. Methods

### 3.1. Determination of Combinatorial Effects of MTO and βE

The combinatorial effects of MTO and βE in different ratios (*w*/*w*) were assessed against K562/DOX through MTT assay. With a density of 5 × 10^3^ cells/well, K562/DOX cells were seeded in 96-well plates and incubated for 48 h, then treated with different concentrations of MTO dissolved in the free medium of RPMI-1640, βE dissolved in the mixture of (RPMI-1640 and 0.5% DMSO), and different combination mass ratios of MTO/βE (10:1, 5:1, 2:1, 1:1, 1:2, 1:5, and 1:10 *w*/*w*). After 48 h of incubation, cells were washed twice with cold PBS, and then incubated again for 4 h with 20 µL of MTT solution (5 mg/mL) added to each well. In order to solubilize the formazan crystals, 150 μL of DMSO per well was added to replace the medium. Finally, a Microplate Reader (Biotech, Winooski, VT, USA) was used to measure the UV absorbance intensity at 570 nm. Each measurement was performed in triplicate [29]. The cell viability percentage was evaluated using the following equation:

$$\mathrm{Cell}\;\mathrm{Viability}\;\left(\%\right)=\frac{{\mathrm{OD}}_{\mathrm{sample}\;}}{{\mathrm{OD}}_{\mathrm{control}}}\times 100\%$$

where OD_Sample_ stands for the optical density (absorbance intensity) of the cells treated with MTO, βE, or MTO in combination with βE, while OD_Control_ is the absorbance intensity of the untreated cells. Graph Pad Prism 5.0 software was used to calculate 50% inhibition concentrations (IC_50_) of different individual drugs and their established combinations. All experiments were in triplicate and the results were given as means ±SD. The synergistic effect of different combinations of MTO and βE was assessed through the calculation of combination index (CI) using the equation of Chou and Talalay [30]. The CI was calculated using the following equation:

$$\mathrm{CI}=\frac{{\left(D\right)}_{1}}{{\left(D_{50}\right)}_{1}}+\frac{{\left(D\right)}_{2}}{{\left(D_{50}\right)}_{2}}$$

where (D)_1_ and (D)_2_ are the 50% inhibition concentrations of MTO and βE in combination, while (D_50_)_1_ and (D_50_)_2_ represent the IC_50_ values of MTO and βE alone, respectively. Following the equation, CI < 1 indicates synergism, CI > 1 represents antagonism, and CI = 1 displays additivity.

### 3.2. The Preparation of SLNs

SLNs were prepared via the solvent evaporation method as described by Shi et al. with some modifications [31]. In a 0.5-L round-bottomed flask, different components (250 mg of SPC, 25 mg of trilaurin, 75 mg of cholesterol, 20 mg of S100, 10 mg of mitoxantrone hydrochloride (MTO·HCl) alone and/or 20 mg of βE, 20 mg of SDS) were dissolved in 30 mL of methanol/dichloromethane solvent mixture (1:1, *v*/*v*). Both organic solvents were evaporated using the rotary vacuum evaporator (Haiya Rong Biochemical Instrument, Beijing, China) at 37 °C for 1 h to obtain the lipid film. The lipid film was continued to keep in vacuum for further removing the residual organic solvents for 12 h. Then, the dried lipid film was hydrated with 20 mL of Kolliphor EL solution (2%, *w*/*w*) at 37 °C for 30 min, and the suspension was ultrasonicated at 300 W for 5 min using the Ultrasonic Probe Sonicator (Scientz Biotechnology Co. Ltd., Ningbo, Zhejiang China). The final MTO/βE-SLNs were obtained after filtering twice through a 0.22-µm microfiltration membrane (Millipore, MA, USA) to eliminate non-encapsulated drugs. The MTO-SLNs were also prepared for control. Both MTO/βE-SLNs and MTO-SLNs were stored at 4 °C until further use. For the DiR-loaded MTO/βE-SLN preparations involving the ex vivo imaging study, DiR was incorporated into the nanoparticles whilst dissolved in (1:1, *v*/*v*) methanol/dichloromethane. In brief, 2 mg of DiR was dissolved in 30 mL of organic solvents containing 15 mL of methanol and 15 mL of dichloromethane. Other components as mentioned above were then dissolved in this mixed solvent containing DiR.

### 3.3. The Characterization of SLNs

The particle size, polydispersity index (PDI), and zeta potential of MTO/βE-SLNs and MTO-SLNs were measured using dynamic light scattering (DLS, Zeta plus, Brookhaven, NY, USA) at room temperature. The morphologies of MTO/βE-SLNs and MTO-SLNs were observed using transmission electron microscopy (TEM, Hitachi H7650, Tokyo, Japan) [32,33]. Briefly, samples were placed over a carbon-coated copper grid with some of the particles adhered to the carbon substrate. Then, a drop of 2% phosphotungstic acid solution was placed to act as a negative stain. The samples were left to air-dry before detection.

The amount of MTO and βE encapsulated in SLNs was determined by HPLC. Briefly, 200 μL of MTO/βE-SLNs or MTO-SLNs were dissolved in 10 mL of mobile phase and sonicated for 5 min, followed by centrifugation for 10 min at 12,000 rpm. The supernatant was analyzed by HPLC to determine the total concentration of MTO or βE. The column (waters μBondapak C_18_150 × 4.6 mm, 5 μm) was kept at 40 °C. The mobile phase for MTO consisted of methanol and 0.2 mol/L ammonium acetate aqueous solution (48:52 *v*/*v*). The wavelength detection was monitored at 242 nm with 1.0 mL/min flow rate [32]. The mobile phase of βE was consisted of acetonitrile/water (90:10 *v*/*v*), detected at 210 nm [33]. The injection volume was 20 μL. The following equation was used to calculate the entrapment efficacy (EE %):

$$\mathrm{EE}\;\%=\frac{\mathrm{The}\;\mathrm{amount}\;\mathrm{of}\;\mathrm{encapsulated}\;\mathrm{drug}}{\mathrm{The}\;\mathrm{amount}\;\mathrm{of}\;\mathrm{drug}\;\mathrm{added}}\times 100\%$$

The storage stability of SLNs was performed at 4 °C for three months. The particle size, PDI, the zeta potential, and EE% were used as the main parameters to evaluate the stability of SLNs [34].

### 3.4. Differential Scanning Calorimetry (DSC) Analysis

Thermal Analyzer-60 WS DSC-60 (Shimadzu, Kyoto, Japan) was used to detect the thermal properties of MTO/βE-SLNs [4]. Different samples of MTO, βE, blank SLNs, the physical mixture of MTO, βE, and blank SLNs, and MTO/βE-SLNs were weighed and placed in a sealed aluminum crimp, tested, and heated from 30 °C to 300 °C at a 10 K/min heating rate.

### 3.5. In Vitro Drug Release

The in vitro release was performed using the dialysis bag method (molecular weight cutoff 8–12 kDa) [34,35]. A shaking speed incubator was used to performed dialysis at 37 °C with PBS 1% (*w*/*v*) Tween-80 at pH 7.4 as the release medium. Briefly, an appropriate amount of MTO/βE-SLNs were dispersed in 1 mL PBS (pH 7.4) and placed in dialysis bags, then submerged in 50 mL of release medium. At predetermined time intervals (0.5 h, 1 h, 2 h, 4 h, 6 h, 8 h, 12 h, and 24 h), 2-mL aliquots of the sample were withdrawn, and volumes were replenished with fresh release medium. Upon centrifugation at 12,000 rpm for 5 min, samples were filtered through a 0.22-μm microfiltration membrane (Millipore, MA, USA). The concentrations of MTO and βE in the supernatant were quantified using the HPLC system.

### 3.6. The Cytotoxicity Study of SLNs

The cytotoxicity of blank SLNs was determined by MTT assay [10]. Briefly, K562 cells and K562/DOX cells were seeded in 96-well plates at a density of 5 × 10^3^ cells/well and cultured for 48 h. The cells were incubated with different concentration of blank SLNs (10, 25, 50, 100, 200, 400, and 500 μg/mL). Afterward, 20 μL of MTT solution (5 mg/mL) was added to each well, followed by 4 h incubation at 37 °C. The medium was replaced with 150 μL of DMSO to dissolve the formazan crystals. Finally, the absorbance was determined at 570 nm using a Microplate Reader (Biotech, Winooski, VT, USA).

A cytotoxicity study of MTO, MTO/βE, MTO-SLNs, and MTO/βE-SLNs was also carried out by MTT assay in both K562 and K562/DOX cells lines. K562 cells and K562/DOX cells were seeded in a 96-well plate at a density of 5 × 10^3^ cells/well and incubated for 48 h. Then, the cells were treated with MTO, MTO/βE, MTO-SLNs, and MTO/βE-SLNs at different doses (total amount of MTO in SLNs: 0.01, 0.1, 1, 2.5, 5, and 10 μg/mL in K562 cells and 0.1, 1, 10, 20, 40, and 80 μg/mL in K562/DOX cells for 48 h. After 48 h of incubation, cells were washed twice with cold PBS then incubated again for 4 h with 20 µL of MTT solution (5 mg/mL) added to each well. In order to solubilize the formazan crystals, 150 μL of DMSO per well was added to replace the medium. Finally, a Microplate Reader (Biotech) was used to measure the UV absorbance intensity at 570 nm. Each measurement was performed in triplicate. The cell viability percentage was evaluated using the following equation [4,10]:

$$\mathrm{Cell}\;\mathrm{Viability}\;\left(\%\right)=\frac{{\mathrm{OD}}_{\mathrm{sample}\;}}{{\mathrm{OD}}_{\mathrm{control}}}\times 100\%$$

where OD_sample_ stands for the absorbance intensity of the cells treated with MTO, MTO/βE, MTO-SLNs, and MTO/βE-SLNs, while OD_Control_ is the absorbance intensity of the untreated cells. The 50% inhibition concentrations (IC_50_) of different agents were calculated by Graph Pad Prism 5.0 software. All experiments were in triplicate and the results were given as means ± SD.

The different effects of drug formulations on K562/DOX were evaluated in the MDR reversal capacities through the index of drug resistance (IDR) and index of reversal of drug resistance (IRDR) [10]. IDR and IRDR were calculated as follows:

$$\mathrm{IDR}=\frac{{\mathrm{IC}}_{50}\mathrm{of}\;\mathrm{other}\;\mathrm{formulation}\;\mathrm{against}\;K562/\mathrm{DOX}\;\mathrm{cells}}{{\mathrm{IC}}_{50}\mathrm{of}\;\mathrm{MTO}\;\mathrm{against}\;K562\;\mathrm{Cells}}\;\mathrm{IRDR}=\frac{{\mathrm{IC}}_{50}\mathrm{of}\;\mathrm{MTO}\;\mathrm{against}\;K562\;\mathrm{cells}\;}{{\mathrm{IC}}_{50}\mathrm{of}\;\mathrm{other}\;\mathrm{formulation}\;\mathrm{against}\;K562/\mathrm{DOX}\;\mathrm{cells}}$$

### 3.7. Cellular Uptake and Its Mechanism

Flow cytometry was used to investigate the cell uptake with DOX as a fluorescent probe. DOX was selected and replaced MTO due to their similar P-gp efflux MDR mechanism [36,37]. SLNs loaded with DOX and βE (DOX/βE-SLNs) or SLNs loaded with DOX (DOX-SLNs) were prepared using the same method as for MTO/βE-SLNs and MTO-SLNs. K562 cells and K562/DOX cells were seeded in 24-well plates (Costar, Washington, DC, USA) at a density of 5 × 10^4^ cells/well and incubated for 24 h. In order to evaluate time and concentration effects on cell uptake, the incubated medium was replaced with DOX, DOX/βE, DOX-SLNs, and DOX/βE-SLNs. Different concentrations of 1, 5, 10, 20 μg/mL of DOX (treated for 4 h) were used to assess the concentration effect, and incubation times of 0.5, 1, 2, 4, and 8 h (at 5 µg/mL of DOX) were used to evaluate the time effect. Ice-cold PBS was used to wash the cells three times after incubation. The cells were collected by centrifugation at 1000 rpm for 3 min and analyzed by flow cytometry (BD Biosciences, San Jose, CA, USA) [4].

To confirm the internalization mechanism of DOX/βE-SLNs in K562 and K562/DOX, the cells were seeded in 24-well plates (5 × 10^4^ cells/well) for 24 h. Then, the culture medium was replaced with fresh medium containing different inhibitors including chlorpromazine (10 µg/mL), nystatin (10 µM), M-β-CD (2.5 mM), and amloride (0.1 mM). After saturating with inhibitors for 30 min at 37 °C, the cells were incubated for 4 h with DOX/βE-SLNs at 5 μg/mL of DOX. Finally, cells were collected, and the fluorescence intensity was measured by flow cytometry [38].

### 3.8. Drug Efflux Study

In order to determine DOX efflux, K562 and K562/DOX cells were seeded and incubated in 12-well plates (1 × 10^5^ cells/well). Different DOX formulations diluted with fresh medium to a concentration of 10 μg/mL were incubated with K562 and K562/DOX cells for 4 h at 37 °C. Subsequently, the cells were washed three times with ice-cold PBS, and then incubated with free medium at another 0.5, 1, 2, and 4 h. Ice-cold PBS was used to wash the cells at the end of each incubation time. The amounts of DOX retained in cells were determined by flow cytometry. The investigation was carried out in triplicate [10].

### 3.9. Intracellular ATP Production Assay

An ATP assay kit was used to measure the intracellular ATP. K562/DOX cells at a density of 1 × 10^5^ cells/well were seeded and cultured in 12-well plates and incubated for 24 h. The cells were then incubated with fresh medium containing MTO, MTO/βE, MTO-SLNs, and MTO/βE-SLNs for 8 h at 37 °C. The cell ATP content was determined based on the manufacturer’s protocol. Briefly, the cells were collected and lysed by 200 μL of lysis solution to obtain the cell lysate, followed by centrifugation at 12,000× *g* for 5 min at 4 °C. Next, 20 μL of the supernatant of each sample was mixed with 100 μL of ATP testing solution in a black 96-well plate, and kept for 10 min. A microplate luminometer (Luminoskan, Thermo Scientific, Waltham, MA, USA) was used to measure the luminescence intensity. The raw data were converted to ATP concentrations using the standard ATP calibration [38].

### 3.10. Pharmacokinetic and Biodistribution Studies

The pharmacokinetics of MTO, βE, and MTO/βE-SLNs were determined in male SD rats (body weight, 180–220 g). All the preparations were administered intravenously into the tail vein at a dose of 2 mg/kg (*n* = 6) for MTO and 4 mg/kg (*n* = 6) for βE. The blood samples were taken with a heparinized syringe at 0.5, 1, 2, 4, 6, 8, 12, and 24 h. Once withdrawn, 4000 rpm was the speed used to centrifuge the samples for 10 min at 4 °C. Kinetica 4.4 (Thermo Electron Corporation, Waltham, MA, USA) was used to analyze the plasma concentrations versus time data. Furthermore, MTO/βE-SLN biodistribution was determined through in vivo fluorescence imaging experiments. DiR, a hydrophobic near-infrared dye, was loaded into SLNs.

K562/DOX tumor-bearing mice were injected with free DiR and DiR-loaded MTO/βE-SLNs via the tail vein at a dose of 1 mg/kg of DiR. At the time points of 3, 6, 12, and 24 h post injection, the images were taken to observe the biodistribution using an in vivo imaging system. After 24 h post injection, the mice were euthanized, and their major organs (brain, kidney, lung, spleen, liver, heart, and tumor) were harvested for ex vivo imaging. The mean fluorescence intensity of the collected samples was also measured [35].

### 3.11. In Vivo Antitumor Activity

Mice bearing K562/DOX xenografts tumors were used to evaluate different MTO formulations through an in vivo antitumor efficacy study. K562/DOX cells at a concentration of 1 × 10^7^ cells/mice with a volume of 200 μL were injected into the left axilla of BALB/c nude mice. After inoculation of cells, the growth of tumor was observed every two days by measuring the diameter of tumor with Vernier caliper. The tumor volume was calculated as (L × W^2^)/2, where L and W are the tumor length and width, respectively. The anti-tumor evaluation was started when tumor volume reached about 100 mm^3^, and this day was designated as day 0. Mice were randomly divided in five groups of six: Saline, MTO, MTO/βE, MTO-SLNs, and MTO/βE-SLNs (MTO dosage: 2 mg/kg and βE dosage: 4 mg/kg). The injections were carried out on days 0, 3, 6, 9, and 12 via the tail vein. In order to evaluate the treatment efficacy and safety, mice were sacrificed, and then tumors, heart, lung, liver, kidney, and spleen were dissected and fixed in 4% paraformaldehyde. In order to observe the pathological changes in different tissues, samples were cut and stained with hematoxylin and eosin (H&E) for histological analyses. An optical microscope (Leica DMI6000 B, Wetzlar, Germany) was used to take different photos. In addition, TdT-mediated dUTP nick-end labeling analysis (TUNEL) was also conducted in the tumor sections to further confirm the anti-cancer function of different preparations [35,38].

### 3.12. Statistical Analysis

All experimental data with SPSS 11.0 software (SPSS Inc., Chicago, IL, USA) were analyzed by one-way analysis of variance (ANOVA) and Student’s *t*-test. The differences were considered to be significant at *p* < 0.05 and *p* < 0.01.

## 4. Results and Discussion

### 4.1. Combinatorial Effects of MTO and βE

Drugs combinations can produce a synergistic effect, which are above the expected potencies and efficacies of individual drugs. However, some combinations showed sub-additive or simple additive effects. Thus, in combination drug delivery, it is most important to quantitatively determine the therapeutic effects in terms of synergistic or additive effects [30]. The final proportion of the drugs combined is crucial [39]. The toxicity of the MTO and βE combination toward K562/DOX cells was investigated in different mass ratios (*w*/*w*). The IC_50_ values of free MTO, free βE, and the combinations of MTO and βE (MTO/βE = 10:1, 5:1, 2:1, 1:1, 1:2, 1:5, and 1:10, *w*/*w*) after 48 h of incubation are shown in Table 1.

All the combined drugs exhibited lower IC_50_ values relative to free MTO. This outcome indicates improvement in the cytotoxic effects against K562/DOX cells based on the combined ratios of MTO and βE. The CI values of all the weight ratio combinations lower than 1 indicate synergism due to MTO and βE combination. The combination of MTO and βE in a weight ratio of 1:2 (*w*/*w*) exhibited the lowest CI value of 0.38 after 48 h of incubation; hence, it was used for further investigation.

### 4.2. Characterization of SLNs

SLNs were prepared by solvent evaporation method. An ion-pairing strategy using SDS to neutralize the charge increased their affinity with the oil phase in the core. With the formation of the ion–pair complex, the positively charged MTO separated to a higher degree in the hydrophobic core and was successfully entrapped in the SLNs. In this study, MTO and βE in the weight ratio of 1:2 were co-encapsulated in SLNs. As shown in Table 2, the particle sizes were 121.5 ± 1.8 and 124.6 ± 1.4 nm for MTO-SLNs and MTO/βE-SLNs, respectively, assessed by dynamic light scattering (DLS) with polydispersity indices of less than 0.20. The particle size is of utmost importance because it determines endocytic characteristics of tumor cells and it also affects the buildup of nanoparticles in tumor sites through the enhanced permeability and retention (EPR) effect [40].

**Figure 1:**
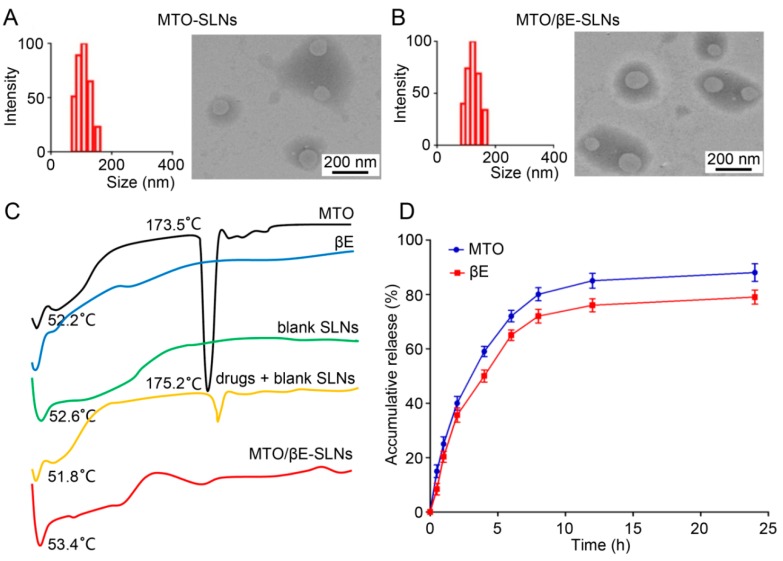
(**A**,**B**) The particle size and TEM images of (**A**) MTO-SLNs and (**B**) MTO/βE-SLNs. (**C**) The differential scanning calorimetry (DSC) analysis of MTO powder, βE, blank SLN powder, physical mixture of drugs (MTO/βE) and blank SLNs powder, MTO/βE-SLNs powder. (**D**) The in vitro release of MTO and βE from MTO/βE-SLNs at pH 7.4. Data represent means ± SD (*n* = 3). ANOVA performed for the release profile of MTO and βE after 24 h reveals a statistically reliable difference at 95% confidence. The accumulated release of MTO was observed to be higher than that of βE after 24 h. Both MTO and βE exhibited a similar release tendency of the drug in this study.

Furthermore, the TEM images revealed that MTO-SLNs and MTO/βE-SLNs possessed approximately spherical morphology and uniform size distribution (Figure 1A,B). A similar particle size was found between DLS and TEM. The zeta-potential of the prepared SLNs ranged from −20.86 to −16.47 mV. It was reported that a slightly negatively nanoparticle could decrease reticuloendothelial system (RES)-mediated clearance, and eventually increase circulation time [41]. MTO and βE were efficiently loaded within SLNs, and the EE% of all the prepared SLNs was higher than 90%. The ideal EE% of MTO was mainly due to the formation of an ion–pair complex between SDS and positively charged MTO, while, for βE, the loaded mechanism was due to the hydrophobic interaction between βE and lipid core of SLNs. The MTO-SLNs and MTO/βE-SLNs were found to be stable at 4 °C for three months as evidenced by the almost intact particle size, PDI, zeta potential, and EE% values (Table 2).

The physical status of drugs in SLNs and interaction between drugs and the lipid matrix should be investigated when developing SLN formulations [42,43]. From the results of DSC analysis in Figure 1C, the endothermic peaks of all samples between 50 °C and 60 °C were due to the loss of absorbed and bonded water. Pure MTO showed a sharp endothermal peak at 173.5 °C, which was the typical behavior of MTO [44], while the thermograms of βE did not show any characteristic peak due to its liquid state. The thermograms of the physical mixture of drugs and blank SLNs showed a low-intensity characteristic peak of MTO, which would be due to the interaction between blank SLNs with the two drugs during the mixing process. This phenomenon was earlier noticed for solid dispersions made with a hydrophilic carrier [45]. There was no significant difference in the diffraction pattern of blank SLNs and MTO/βE-SLNs, indicating that the loading of MTO and βE did not change the nature of SLNs and the molecular or amorphous state of the drugs entrapped.

The in vitro release profiles of MTO and βE from MTO/βE-SLNs are shown in Figure 1D. The results show that MTO and βE exhibited similar release patterns. MTO/βE-SLNs displayed a sustained drug-release profile of both drugs, and approximately 89% of MTO and 78% of βE were released from MTO/βE-SLNs at 24 h. This similarity in the pattern of drug release could be of benefit in the synergistic nature of MTO and βE co-delivery.

### 4.3. In Vitro Cytotoxicity Studies

**Figure 2:**
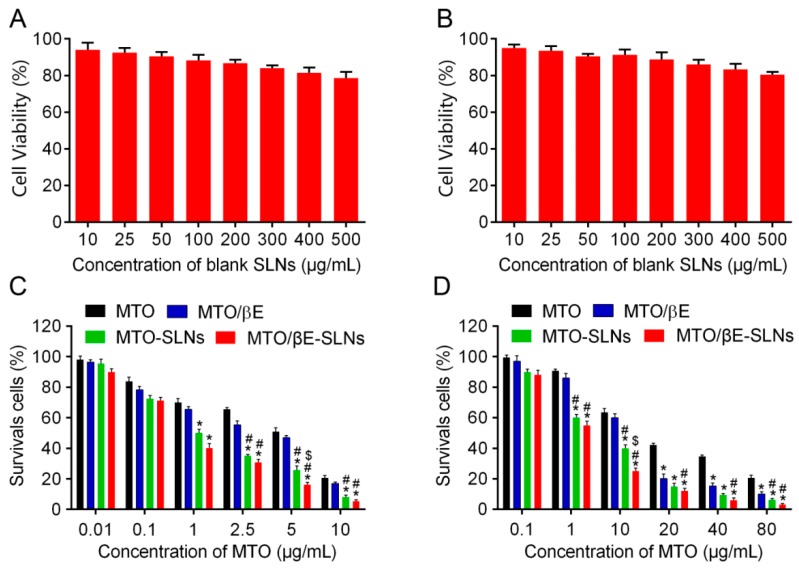
(**A**) In vitro cytotoxicities of blank SLNs in K562 cells after 48 h of incubation. (**B**) In vitro cytotoxicities of blank SLNs in K562/DOX cells after 48 h of incubation. (**C**) In vitro cytotoxicities of MTO, MTO/βE, MTO-SLNs, and MTO/βE-SLNs in K562 cells after 48 h of incubation. (**D**) In vitro cytotoxicities of MTO, MTO/βE, MTO-SLNs, and MTO/βE-SLNs in K562/DOX cells after 48 h of incubation. Data represent means ± SD (*n* = 3). * *p* < 0.05 versus MTO; ^#^ *p* < 0.05 versus MTO/βE; ^$^ *p* < 0.05 versus MTO-SLNs.

The cell viability of K562 cells and K562/DOX cells incubated with blank SLNs at tested concentrations for 48 h were above 80% (shown in Figure 2A,B), suggesting that blank SLNs might be good biocompatible carriers for drug delivery.

The cytotoxicities of drug-free and drug-loaded SLNs were determined to evaluate their impact at decreasing drug resistance. As indicated in Figure 2C,D, all the preparations showed concentration-dependent inhibition of K562 cells and K562/DOX cells. No cytotoxicity against K562 cells and K562/DOX cells was found when the concentration of MTO was less than 0.1 μg/mL. The cell survival rate of K562/DOX cells was reduced to 29.81% when the concentration of MTO was 10 μg/mL in MTO/βE-SLNs, while the survival rate of K562/DOX cells was 81.43%, 62.27%, and 46.57% when treated with MTO, MTO/βE, and MTO-SLNs, respectively, suggesting that the cytotoxicity of MTO/βE-SLNs was significantly higher than that of the other three groups at the same concentrations. When the concentration of MTO increased to 80 μg/mL, the survival ratio of MTO/βE-SLNs, MTO-SLNs, MTO/βE, and MTO decreased to 3.31%, 7.28%, 13.89%, and 24.06% in K562/DOX cells, respectively. The results of IC_50_ revealed that K562 cells were sensitive to free MTO (IC_50_ of 5.02 μg/mL), while K562/DOX cells were resistant to free MTO (IC_50_ of 48.19 μg/mL) (Table 3). In addition, MTO/βE exhibited slightly better cytotoxicity than MTO toward K562 cells (IC_50_ of 1.82 μg/mL), but could reverse the MDR of K562/DOX cells with IC_50_ of 11.01 μg/mL. Furthermore, MTO-SLNs showed better cytotoxicity than MTO/βE in K562 cells (IC_50_ of 0.98 μg/mL) and K562/DOX cells (IC_50_ of 5.61 μg/mL, *n* = 3). After loading MTO and βE into SLNs, MTO/βE-SLNs showed greater cytotoxicity in both K562 cells (IC_50_ of 0.51 μg/mL) and K562/DOX cells (IC_50_ of 2.25μg/mL) when compared with MTO-SLNs, free MTO/βE, and MTO. The enhanced cytotoxicity would be due to the inhibition of P-gp by βE [15,20], which could improve the accumulation of released MTO in K562/DOX cells. To further illustrate the overcoming of the MDR effect, both IDR and IRDR was calculated. The results showed that IDR decreased from 9.59 for MTO to 6.05, 5.72, and 4.41 for MTO/βE, MTO-SLNs, and MTO/βE-SLNs, while the IRDR was increased from 1.58, 1.67, and 2.17 for MTO/βE, MTO-SLNs, and MTO/βE-SLNs, respectively (Table 3). It is worth noting that MTO/βE-SLNs exhibited a stronger ability in reversing drug resistance compared to other formulations, as confirmed by the higher IRDR and the lower IDR.

### 4.4. Cellular Uptake Studies

To study the uptake behavior of different SLNs in K562 and K562/DOX cells, cells were treated with DOX, DOX/βE, DOX-SLNs, and DOX/βE-SLNs at different times and concentrations, where DOX was used as a fluorescent probe for flow cytometry. The particle sizes and zeta potentials of DOX-SLNs and DOX/βE-SLNs, and the EE% of DOX were 130.5 ± 1.9 nm and 132.1 ± 1.3 nm, −22.26 ± 1.2 mV and −20.18 ± 1.6 mV, 98.49% ± 1.24% and 96.17% ± 1.81%, respectively, with polydispersity indices of less than 0.25. The EE% of βE in DOX/ βE-SLNs was 93.35% ± 1.56%. Furthermore, DOX/βE-SLNs displayed a sustained drug-release profile of both drugs (DOX and βE) and 84% of DOX and 76% of βE released within 24 h, which was similar to the release of MTO and βE from MTO/βE-SLNs (89% and 78%). These similarities of both drugs (MTO and DOX) in the pattern of drug release could be beneficial in the uptake studies.

**Figure 3:**
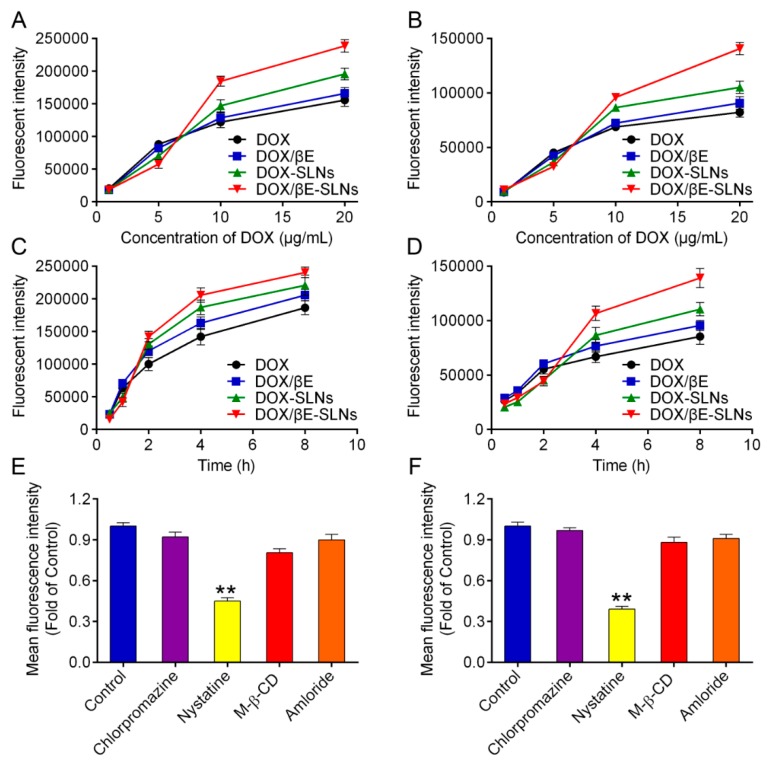
(**A**,**B**) Uptake of DOX, DOX/βE, DOX-SLNs, and DOX/βE-SLNs into K562 cells (**A**) and K562/DOX cells (**B**) when treated with different concentration of DOX for 4 h. (**C**,**D**) Uptake of DOX, DOX/βE, DOX-SLNs, and DOX/βE-SLNs into K562 cells (**C**) and K562/DOX cells (**D**) at different time points. (**E**,**F**) The internalization mechanism for DOX/βE-SLNs in K562 cells (**E**) and K562/DOX cells (**F**) after treating with different inhibitors. Measurements were performed in triplicate. Data represent means ± SD (*n* = 3). ** *p* < 0.01 versus control group.

Flow cytometry was used to quantify the extent of cellular uptake for the various DOX formulations. As shown in Figure 3A,B, a clear concentration-dependent internalization of DOX, DOX/βE, DOX-SLNs, and DOX/βE-SLNs in K562 cells and K562/DOX cells can be observed. The fluorescent intensity of DOX-SLNs and DOX/βE-SLNs was significantly enhanced as the concentration of DOX was 10 μg/mL and 20 μg/mL. In addition, the uptake of DOX by K562/DOX cells was much lower than that by K562 cells, which might be due to the obvious efflux of DOX by K562/DOX cells. SLNs co-loaded with DOX and βE could improve K562/DOX cell uptake of DOX significantly when compared with DOX-SLNs, suggesting that the βE could reduce the extracellular efflux of K562/DOX cells. Furthermore, either K562 cells or K562/DOX cells showed a time-dependent cellular uptake (Figure 3C,D). The uptake curves of DOX-SLNs or DOX/βE-SLNs increased quickly after 2 h of incubation. The total uptake concentration of DOX/βE-SLNs at 8 h in K562 cells and K562/DOX cells was significantly higher than that of other groups.

To further confirm the uptake internalization mechanism of DOX/βE-SLNs in K562 cells and K562/DOX cells, different endocytosis inhibitors were employed to block the nanoparticle entry into the cells, such as chlorpromazine (catherin-mediated endocytosis), nystatin (calveolin-mediated endocytosis), M-β-CD (lysosome-involved endocytosis), and amloride (macropinocytosis endocytosis) [46,47,48]. As shown in Figure 3E,F, the fluorescence intensity of DOX/βE-SLNs was significantly reduced when pretreated with nystatin in K562 cells and K562/DOX cells, which suggested that DOX/βE-SLNs enter into K562 cells and K562/DOX cells through calveolin-mediated endocytosis.

### 4.5. In Vitro Drug Efflux and Intracellular ATP Production Assay

**Figure 4:**
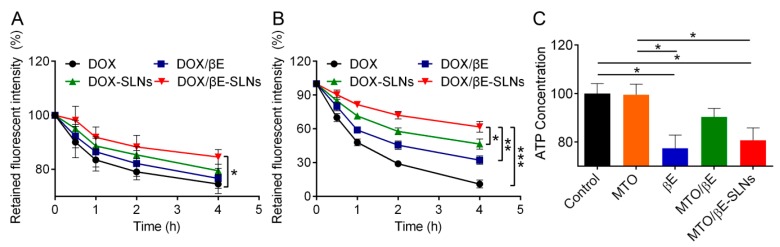
(**A**,**B**) The amount of DOX retained in the K562 cells (**A**) and K562/DOX cells (**B**) after treating with DOX, DOX/βE, DOX-SLNs, and DOX/βE-SLNs for 4 h at 37 °C. (**C**) The amount of intracellular ATP in K562/DOX cells after treatment with MTO, βE, MTO/βE, and MTO/βE-SLNs. Data are presented as means ± SD, *n* = 3. * *p <* 0.05, ** *p <* 0.01, and *** *p <* 0.001.

To evaluate in vitro drug efflux, K562 cells and K562/DOX cells were firstly cultured with DOX, DOX/βE, DOX-SLNs, and DOX/βE-SLNs, following the protocol stated earlier. As shown in Figure 4B, about 90%, 69%, 54%, and 38% of DOX underwent efflux in the groups of DOX, DOX-βE, DOX-SLNs, and DOX-βE-SLNs by K562/DOX cells, respectively. The highest efflux rate as evidenced by the DOX was significantly reduced in the DOX/βE due to the inhibition of P-gp by βE. There was a further reduction in efflux rates in the co-encapsulated DOX/βE-SLNs. In contrast, for K562 cells, the concentration of DOX was markedly higher due to the reduced expression of P-gp, resulting in negligible drug efflux rate for all the DOX formulations (Figure 4A). It was reported that P-gp, a well-studied drug efflux transporter, belongs to the ATP-binding cassette (ABC) super family. This transporter use energy obtained from the hydrolysis of ATP to remove or efflux various endogenous ligands such as proteins, lipids, metabolic products, and drugs such as cytotoxic and antibiotics from cancer cells, thereby significantly lowering their intracellular drug concentrations and attenuating their efficacy [49].

Therefore, the effect on intracellular ATP by MTO, βE, MTO/βE, and MTO/βE-SLNs was examined. βE, MTO/βE, and MTO/βE-SLNs displayed significant inhibitory capacity toward ATP activity in K562/DOX cells (Figure 4C). The decrease in intracellular ATP production would induce the inactivation of the P-gp efflux transporter [50]. Thus, the reversed MDR mechanism of MTO/βE-SLNs might also be ascribed to the inhibition of intracellular ATP production by βE.

### 4.6. The Studies of Pharmacokinetics and Biodistribution of MTO/βE-SLNs

**Figure 5:**
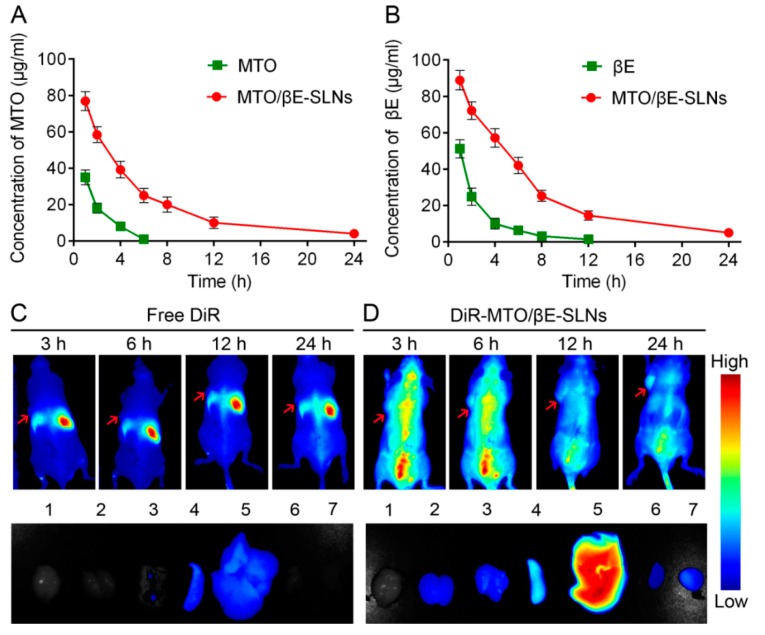
(**A**) The drug plasma concentration–time curve of MTO after an intravenous injection of free MTO and MTO/βE-SLNs at a dose of 2 mg/kg. (**B**) The drug plasma concentration–time curve of βE after an intravenous injection of free βE and MTO/βE-SLNs at a dose of 4 mg/kg. Measurements were performed, and data are presented as means ± SD (*n* = 6). (**C**) In vivo biodistribution of free DiR and ex vivo imaging of excised organs and tumor after 24 h post injection of free DiR in K562/DOX tumor-bearing nude mice. 1: brain; 2: kidney; 3: lung; 4: spleen; 5: liver; 6: heart; 7: tumor. (**D**) In vivo biodistribution of DiR-MTO/βE-SLNs and ex vivo imaging of excised organs and tumor after 24 h post injection of DiR-MTO/βE-SLNs in K562/DOX tumor-bearing nude mice. 1: brain; 2: kidney; 3: lung; 4: spleen; 5: liver; 6: heart; 7: tumor.

For pharmacokinetics studies, MTO, βE, and MTO/βE-SLNs were injected via tail vein into SD rats. It was observed that MTO/βE-SLNs significantly altered the pharmacokinetics of MTO and βE (Figure 5A,B). In the case of free MTO, because of its small molecular weight and rapid elimination, the plasma concentration decreased rapidly and no drug concentrations were observed after 6 h post injection, while, for free βE, the plasma concentration also decreased quickly and no drug signal was obtained after 12 h post injection. After loading MTO and βE into SLNs, the elimination from circulation significantly reduced. From the pharmacokinetic parameters, shown in Table 4, compared with free MTO and βE, the *T*_1/2_ of MTO and βE in MTO/βE-SLNs increased about 8.5- and 5.7-fold, respectively; the area under the curve (AUC_0–24_) of MTO and βE in MTO/βE-SLNs increased about 43.7- and 13.3-fold, respectively. The pharmacokinetic data suggested that MTO/βE-SLNs significantly prolonged the circulation time of both MTO and βE, which would benefit the accumulation of drugs into tumors, thereby necessarily achieving an enhanced drug efficacy.

To further investigate the biodistribution of MTO/βE-SLNs in vivo, DiR-loaded MTO/βE-SLNs were prepared (DiR-MTO/βE-SLNs) and injected into the K562/DOX tumor-bearing nude mice via intravenous injection. As shown in Figure 5C,D, more DiR-MTO/βE-SLNs could accumulate in the tumor site when compared with free DiR, and the fluorescence signal decreased as time prolonged. In addition, the ex vivo image results also revealed that DiR-MTO/βE-SLNs showed a higher fluorescence signal in tumor when compared to free DiR, which would be due to the enhanced blood circulation time and the EPR effect. This result would also be beneficial for constantly inactivating the P-gp drug efflux pump, which would increase the accumulation of βE in the tumor site, thereby increasing the antitumor effects of the cytotoxic MTO drug in MDR tumors.

### 4.7. In Vivo Antitumor Activity

**Figure 6:**
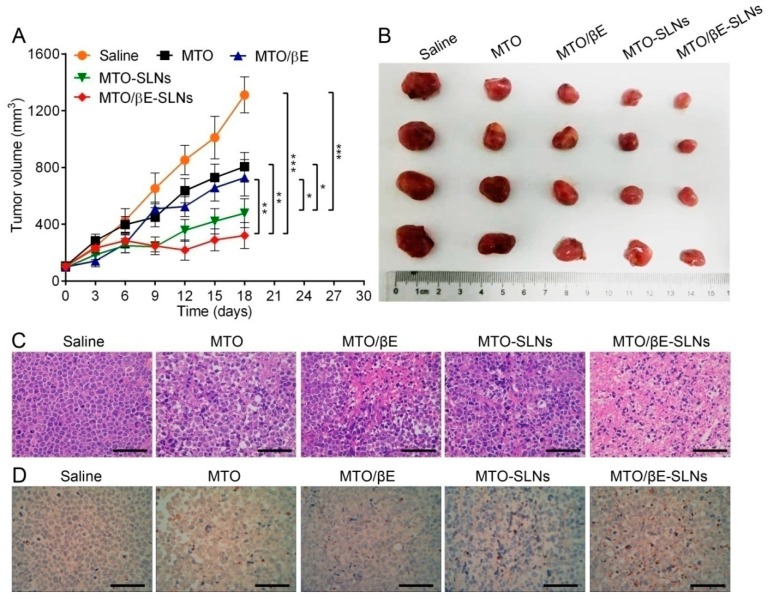
In vivo antitumor efficacies of various MTO formulations in K562/DOX tumor-bearing nude mice. (**A**) The tumor volume changes during the treatment with various formulations. Data are presented as means ± SD, *n* = 5. * *p <* 0.05; ** *p <* 0.01;*** *p <* 0.001. (**B**) The photograph of excised tumors harvested from each group at the end of the experiment. (**C**) Hematoxylin and eosin (H&E) assays of tumors from K562/DOX tumor xenografts-bearing BALB/c nude mice after being subjected to different treatments for 18 days. Scale bar = 200 μm. (**D**) TdT-mediated dUTP nick-end labeling (TUNEL) assays of tumors from K562/DOX tumor xenografts-bearing BALB/c nude mice after being given different treatments for 18 days. Scale bar = 200 μm.

The anti-tumor results showed that MTO/βE-SLNs exhibited the highest antitumor activity when compared with other MTO formulations (Figure 6). The tumor volumes of MTO/βE-SLNs groups were smaller than those of saline, MTO, MTO/βE, and MTO-SLNs in the whole measuring period (Figure 6A,B). MTO and MTO/βE groups showed similar tumor inhibition, which was different from the in vitro cytotoxicity. This phenomenon was due to the fast elimination of MTO and βE in circulation and the insufficient accumulation of MTO and βE in the tumor. After loading MTO into SLNs, the tumor inhibition effect of MTO-SLNs and MTO/βE-SLNs significantly increased. Moreover, the tumor inhibition rate of MTO/βE-SLNs was 75.58% which exhibited the strongest effect compared with other groups. This could be attributed to the consistency of MTO and βE accumulation in the tumor via the EPR effect. The superior anti-tumor effect of MTO/βE-SLNs might also be attributed to the sufficient cellular uptake and similar release kinetics. To further evaluate the efficacy of the various formulations, the BALB/c nude mice were sacrificed, and tumor sections were analyzed via hematoxylin and eosin (H&E) and TUNEL staining. The outcome of these analyses showed varied levels of necrosis, where MTO/βE-SLNs had much larger necrosis regions compared with MTO-SLNs, MTO/βE, and MTO (Figure 6C). Moreover, from the TUNEL images, MTO/βE-SLNs groups could induce more K562/DOX cells to undergo apoptosis, which demonstrated a higher anti-tumor activity (Figure 6D). These results showed the superior efficacy of MTO/βE-SLNs over the other MTO formulations, and they were consistent with the results of the in vitro cytotoxicity study. In addition, after administration of free MTO, the body weight of mice decreased remarkably, while no significant difference was found in body weight between MTO/βE-SLNs and saline (Figure S2, Supplementary Materials). Meanwhile, no obvious damage was found on major organs after administration of MTO/βE-SLNs (Figure S3, Supplementary Materials), suggesting the potential of MTO/βE-SLNs to overcome MDR in leukemia with high biocompatibility.

## 5. Conclusions

The overall goal of this work was to combine and successfully load MTO (anticancer drug) and βE (P-gp inhibitor agent) into SLNs with a precise weight ratio of 1:2 (*w*/*w*), which displayed the highest synergistic effect. Optimized nanoparticles (MTO/βE-SLNs) possessed suitable particle size, negative zeta potential, high drug loading, and good colloid stability. Importantly, the cytotoxicity, cellular uptake, and significant reversal of drug resistance in K562/DOX cells of MTO/βE-SLNs were significantly improved compared to other MTO formulations. The pharmacokinetics study in SD rats showed significantly prolonged circulation time of MTO and βE in MTO/βE-SLNs, which was beneficial for reversion of MDR by constant inhibition of the drug efflux pump. Moreover, the in vivo biodistribution and ex vivo organ images represented enhanced blood circulation time and efficient tumor accumulation of DiR loaded in the SLNs. The in vivo tumor study confirmed that MTO/βE-SLNs could significantly accumulate in the tumor and achieve enhanced drug efficacy for inhibiting the K562/DOX tumor. Additionally, H&E staining and the TUNEL immunohistochemical assay of tumor tissues confirmed the bio-safety and the potent apoptotic activity of MTO/βE-SLNs. These findings suggest that MTO/βE-SLNs might provide a potential therapeutic strategy for leukemia with MDR.

## Figures and Tables

**Table 1:** The half maximal inhibitory concentration (IC_50_) and combination index (CI) of various MTO and βE formulations against K562/DOX cells after 48 h of incubation (*n* = 3).

Sample	IC_50_ (µg/mL)		CI
	MTO	βE	
Free MTO	48.19	-------	-------
Free βE	-------	140.30	-------
MTO/βE (10/1)	38.60	0.39	0.83
MTO/βE (5/1)	32.63	6.53	0.72
MTO/βE (2/1)	24.07	12.04	0.59
MTO/βE (1/1)	15.99	15.99	0.45
MTO/βE (1/2)	11.01	22.02	0.38
MTO/βE (1/5)	8.98	44.90	0.51
MTO/βE (1/10)	8.21	82.08	0.76

CI_50_: combination index; CI < 1 indicates synergism, CI > 1 indicates antagonism, and CI = 1 indicates additivity. IC_50:_ The 50% inhibition concentrations; MTO: mitoxantrone; βE: β-elemene; MTO/βE: MTO and βE drug free combination. The IC_50_ values of MTO in the combination of MTO/βE were statistically analyzed. Analysis of variance (ANOVA) performed at 95% confidence interval revealed that there was a statistically significant difference between IC_50_ values for the various drugs formulations, with *p* < 0.05. A post hoc test with Turkey honestly significant difference (HSD) revealed details of the ANOVA. There were significant differences at every level of comparison except for between MTO/βE (1/5) and MTO/βE (1/10). Similarly, the IC_50_ values of βE in combination were analyzed using ANOVA performed at 95% confidence interval. There was a statistically significant difference between IC_50_ values for the various drug formulations, with *p* < 0.05. A post hoc test with Turkey HSD revealed details of the ANOVA. There were significant differences at every level of the IC_50_ of the drugs used.

**Table 2:** The physicochemical characteristics and the stability at 4 °C for three months of MTO-SLNs and MTO/βE-SLNs (mean ± SD, *n* = 3).

Time (Months)	Formulation	Particle Size (nm)	PDI	Zeta Potential (mv)	EE (%)	
					MTO	Be
0	MTO-SLNs	121.5 ± 1.8	0.171 ± 0.016	−20.86 ± 1.8	99.61	—
	MTO/βE-SLNs	124.6 ± 1.4	0.162 ± 0.011	−16.47 ± 0.9	97.98	94.42
3	MTO-SLNs	125.4 ± 2.1	0.159 ± 0.014	−22.15 ± 1.1	96.32	—
	MTO/βE-SLNs	128.8 ± 1.2	0.195 ± 0.009	−18.33 ± 0.8	94.85	90.22

PDI: polydispersity index; EE: encapsulation efficiency; MTO: mitoxantrone; βE: β-elemene; MTO/βE: MTO and βE (1:2) drug free combination; MTO-SLNs: solid lipid nanoparticles loaded with MTO; MTO/βE-SLNs: solid lipid nanoparticles loaded with MTO and βE.

**Table 3:** The IC_50_, index of drug resistance (IDR), and index of reversal of drug resistance (IRDR) of MTO in K562 cells and K562/DOX cells treated with different formulations (*n* = 3).

Formulations	IC_50_		IDR	IRDR
	K562	K562/DOX		
βE	122.2	140.3	-------	------
MTO	5.025	48.19	9.59	------
MTO/βE	1.82	11.01	6.05	1.58
MTO-SLNs	0.98	5.61	5.72	1.67
MTO/βE-SLNs	0.51	2.25	4.41	2.17

IC_50:_ 50% inhibition concentration; IDR: index of drug resistance; IRDR: index of reversal of drug resistance. Using K562 cells, the IC_50_ values of the various drugs formulations were analyzed. Analysis of variance (ANOVA) performed at 95% confidence interval revealed that there was a statistically significant difference between the overall IC_50_ for the various drugs formulations, with *p* < 0.05. A post hoc test with Turkey HSD revealed details of the ANOVA. Significant differences were observed whilst comparing βE with MTO, MTO/βE, MTO-SLNs, and MTO/βE-SLNs, as well as between MTO with MTO-SLNs and MTO/βE-SLNs. Similarly, the IC_50_ values for K562/DOX were assessed. ANOVA performed at 95% confidence interval revealed that there was a statistically significant difference between IC_50_ values for the various drug formulations, with overall *p* < 0.05. A post hoc analysis using Turkey HSD revealed that there was no reliably significant difference when MTO/βE was compared with MTO-SLNs and MTO/βE-SLNs.

**Table 4:** Pharmacokinetic parameters of MTO and βE after intravenous administration of MTO, βE, and MTO/βE-SLNs in rats at a dose of 2 mg/kg for MTO and 4 mg/kg for βE (*n* = 6).

Formulation	MTO	βE	MTO in MTO/βE-SLNs	βE in MTO/βE-SLNs
AUC_0–24_ (mg·h·L^−1^)	1.18 ± 0.15	4.86 ± 1.76	51.55 ± 2.19 **	64.46 ± 2.95 **
*T*_1/2_ (h)	1.53 ± 0.33	2.9 ± 0.45	13.13 ± 1.15 *	16.53 ± 1.22 *
*C*_max_ (mg/L)	3.45 ± 0.58	6.68 ± 1.21	67.78 ± 2.89 **	90.15 ± 3.59 **

*C*_max_: maximum plasma concentration; *T*_1/2_: terminal elimination half-life; AUC_0–24_: area under the plasma concentration vs. time curve from time zero to 24; MTO: mitoxantrone; βE: β-elemene; MTO/βE: MTO and βE (1:2) combination; MTO-SLNs: solid lipid nanoparticles loaded with MTO; MTO/βE-SLNs: solid lipid nanoparticles loaded with MTO and βE. Statistically significant differences were found between MTO, βE, and MTO/βE-SLNs groups. Data are presented as means ± SD, *n* = 6. * *p* < 0.05 versus MTO, * *p* < 0.05 versus βE, ** *p* < 0.01 versus MTO, and ** *p* < 0.01 versus βE.

## Supplementary Materials

The following are available online at https://www.mdpi.com/1999-4923/12/2/191/s1, Figure S1: Chemical structures of MTO (A) and βE (B), Figure S2: The body weight changes during the treatment with various formulations, Figure S3: H&E assays of major organs from K562/DOX tumor xenografts-bearing BALB/c nude mice after being received different treatments for 18 days. Scale bar = 200 μm.
