# Supplementary material for: Co-Encapsulation of Mitoxantrone and β-Elemene in Solid Lipid Nanoparticles to Overcome Multidrug Resistance in Leukemia
Source: Pharmaceutics. 2020 Feb 23;12(2):191. doi: 10.3390/pharmaceutics12020191 (PMC7076650; doi:10.3390/pharmaceutics12020191)
Supplement: Supplementary file 1 [file pharmaceutics-12-00191-s001.pdf]

pharmaceutics

MDPI

Supplementary Materials: Co-Encapsulation of Mitoxantrone and β-Elemene in Solid Lipid Nanoparticles to Overcome Multidrug Resistance in Leukemia

Kambere Amerigos Daddy J. C., Minglei Chen, Faisal Raza, Yanyu Xiao, Zhigui Su * and Qineng Ping *

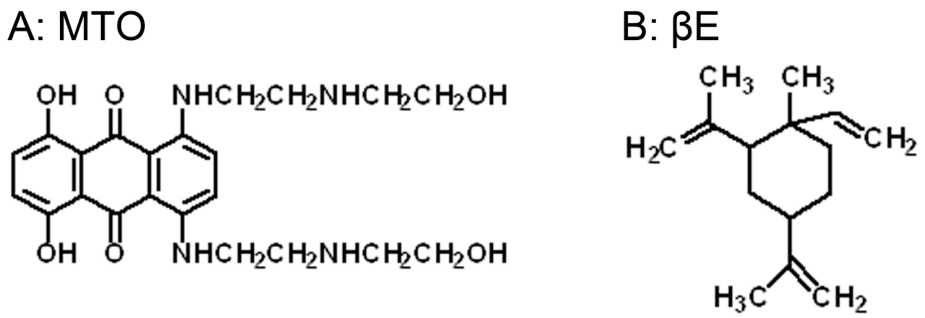

Figure S1. Chemical structures of MTO (**A**) and βE (**B**).

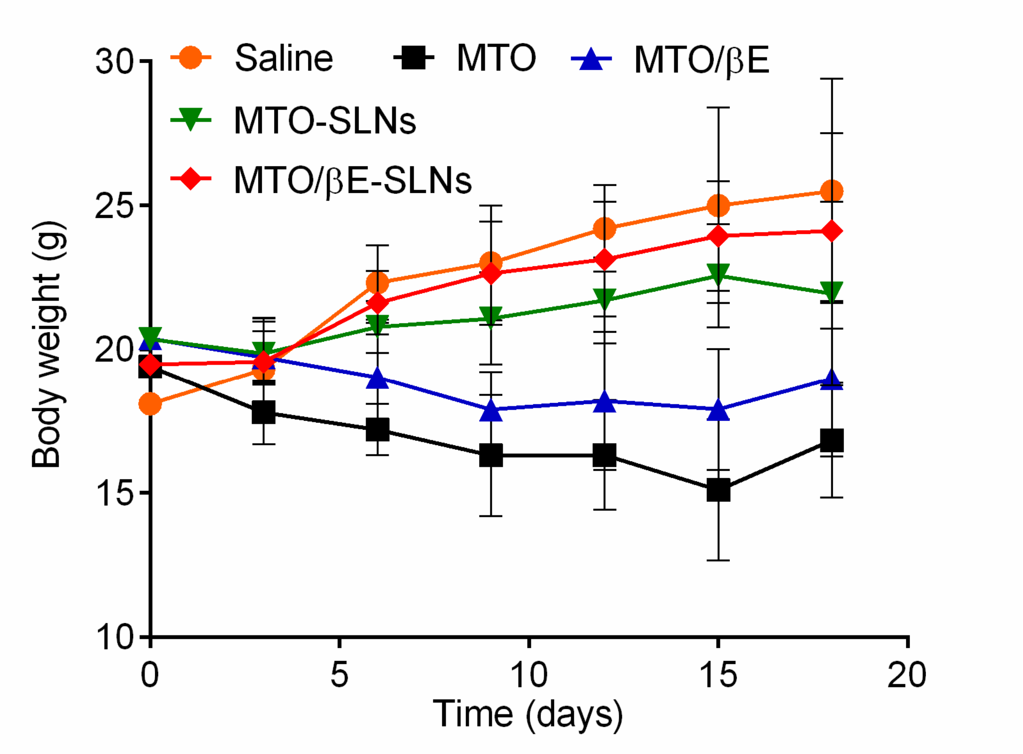

Figure S2. The body weight changes during the treatment with various formulations.

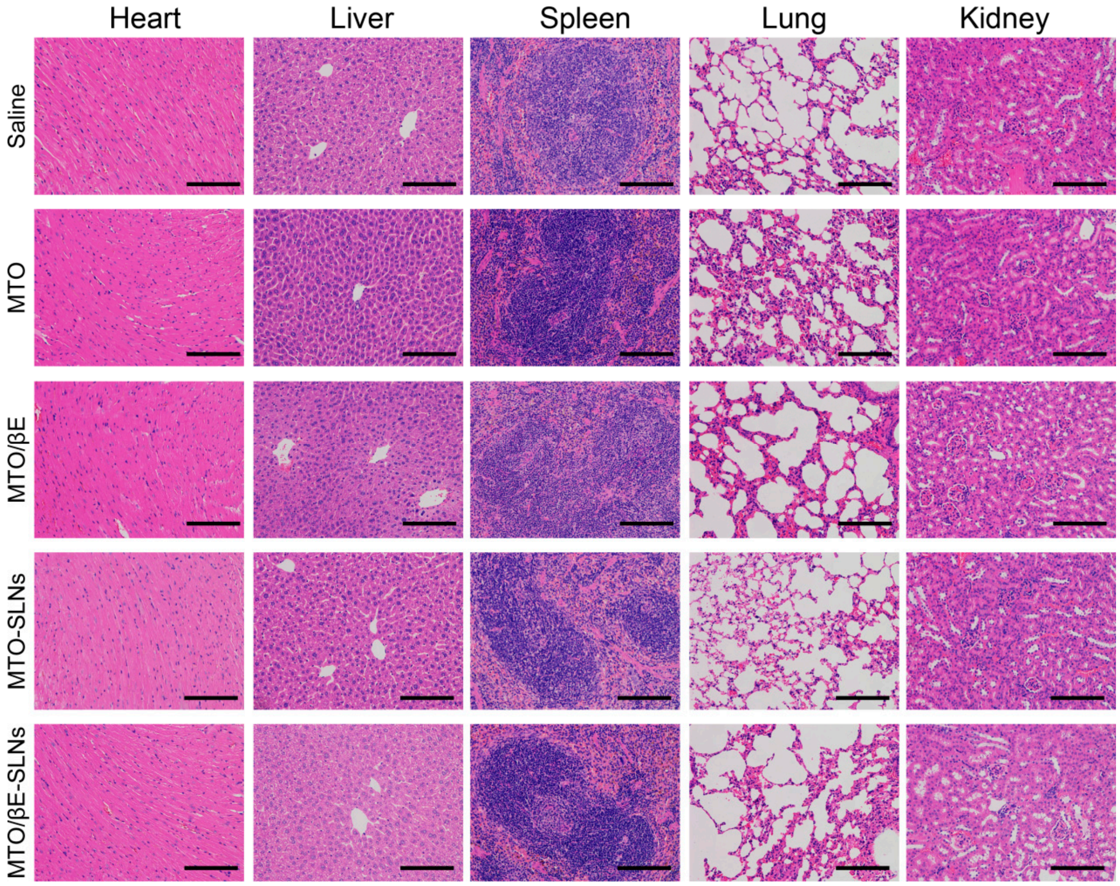

Figure S3. H&E assays of major organs from K562/DOX tumor xenografts-bearing BALB/c nude mice after being received different treatments for 18 days. Scale bar = 200 μm.
